# Outcomes after liposuction-based treatment of lymphedema: a systematic review and meta-analysis

**DOI:** 10.3389/fonc.2025.1651472

**Published:** 2025-11-26

**Authors:** Junzhe Chen, Xiyao Feng, Yan Zhou, Yun Wang, Shune Xiao, Chengliang Deng

**Affiliations:** 1 Department of Burns and Plastic Surgery, Affiliated Hospital of Zunyi Medical University, Zunyi, Guizhou, China; 2 Collaborative Innovation Center of Tissue Repair and Regenerative Medicine, Zunyi, Guizhou, China

**Keywords:** lymphedema, surgery, integrated surgery, liposuction, meta-analysis

## Abstract

**Background:**

Lymphedema, a chronic condition involving lymphatic fluid accumulation, affects over 250 million people worldwide. Liposuction (LS), introduced in 1989, offers a minimally invasive option for non-pitting lymphedema by reducing fibrotic and hypertrophic tissues. However, LS requires ongoing compression therapy as it does not address the underlying lymphatic dysfunction. Although integrated approaches combining LS with lymphovenous anastomosis (LVA) or vascularized lymph node transfer (VLNT) aim to address both fluid removal and lymphatic repair, there remains a lack of consensus regarding the efficacy of these integrated liposuction-based treatments.

**Methods:**

A systematic review and meta-analysis conducted by the PRISMA and AMSTAR guidelines included studies from 1996 to 2024. Fifty-two studies (n=2,334) were reviewed and 23 (n=1,028) were analyzed quantitatively. Outcomes mainly included limb volume reduction, reliance on conservative treatment, improvement in infection rates, and improvement in the quality of life (QOL).

**Results:**

LS-based treatments significantly reduce volume in both upper and lower limbs (91.08% and 92.03%). Standalone LS reduced limb volume by 99.74% but relied on continuous compression therapy. Combined approaches achieved slightly lower reductions (87.31%), but significantly decreased compression dependence, improved lymphatic function, and enhanced QOL. Furthermore, LS-based interventions were associated with a potential reduction in infection episodes, thereby providing long-term benefits.

**Conclusion:**

Liposuction-based therapies effectively manage lymphedema by reducing limb volume and may reduce infections, while improving QOL. In addition, integrated approaches offer additional benefits by directly addressing lymphatic dysfunction and reducing reliance on compression therapy. Standardized methodologies and long-term studies are needed to refine the clinical guidelines and optimize outcomes.

**Systematic review registration:**

https://www.crd.york.ac.uk/PROSPERO, identifier CRD42024616130.

## Introduction

Lymphedema is a condition characterized by abnormal accumulation of lymphatic fluid in the interstitial spaces. Fluid stasis results in progressive tissue swelling, inflammation, and fibrosis (1). Globally, lymphedema affects an estimated 250 million individuals (2), with secondary lymphedema caused by external factors, such as radiation therapy, tumors, infections, or trauma, which are more prevalent than primary lymphedema, which arises from inherent genetic mutations. Importantly, as the incidence of malignant tumors has increased, cancer-related secondary lymphedema has emerged as the most frequent type of lymphedema (3, 4). This condition significantly diminishes the quality of life (QOL) of patients, posing a clinical challenge that requires prompt and effective treatment.

Despite advancements in treatment, challenges persist in managing lymphedema. Clinical strategies can generally be divided into two main types: nonsurgical and surgical. In cases of mild lymphedema, conservative management, especially complex decongestive therapy (CDT) (5), continues to be the preferred treatment. Conversely, moderate to severe instances often necessitate surgical options, which can be classified into two groups: physiological techniques aimed at reestablishing normal lymphatic function and debulking techniques that concentrate on excising excess fibrotic and adipose tissue (6).

Liposuction (LS) is a frequently used debulking technique that was initially introduced for the treatment of upper extremity lymphedema in 1989 (7). Since then, LS has attracted considerable interest as a less invasive alternative to traditional excisional methods, such as the Charles procedure, which necessitates the complete removal of the diseased skin (8). By preserving healthy tissue, LS mitigates tissue damage and decreases the likelihood of complications associated with more invasive surgical options (9). Unlike physiological procedures that focus on reconstructing lymphatic pathways, LS is specifically aimed at eliminating fibrotic and hypertrophic adipose tissue in instances of chronic, non-pitting lymphedema. Numerous studies have demonstrated its effectiveness in achieving significant reductions in limb volume and enhancements in patient-reported QOL (10–12). Nonetheless, variability in study design, such as disparities in follow-up times, patient selection processes, and outcome indicators, restricts the ability to compare these results. Furthermore, LS alone does not address fundamental lymphatic dysfunction, and its long-term effectiveness typically depends on continuous compression therapy. This situation has sparked heightened interest in merging LS with physiological procedures to improve outcomes by addressing both lymphatic drainage and removal of fibroadipose tissue. In the last ten years, the investigation of LS-based integrated (LSI) treatments has expanded markedly. These strategies seek to leverage the complementary interactions between LS and physiological approaches, potentially creating synergistic effects. However, current research on LSIs frequently faces challenges, such as limited sample sizes, brief follow-up periods, and a deficit in standardized methods. These factors impede the establishment of consensus-driven guidelines and restrict the implementation of evidence-based practice.

Despite various systematic reviews and meta-analyses focusing on lymphedema treatments involving LS, the majority have emphasized improvements in quality of life (13), neglecting significant factors, such as volume reduction, reliance on CDT, and infection improvement (14, 15). To date, no systematic review has thoroughly assessed the integration of LS with additional therapeutic approaches (LSIs), and no meta-analysis has identified volume reduction as the primary endpoint. To address these gaps, we conducted a systematic review and meta-analysis examining five key dimensions of LS-based interventions: (1) treatment efficacy, (2) dependence on postoperative compression, (3) infection incidence, (4) quality of life outcomes, and (5) treatment strategy variation and optimization. By gathering and evaluating the latest findings through a systematic review and meta-analysis, this study aims to deliver substantial data that clarifies the role of LS-based treatment in the wider context of lymphedema treatment. Through this methodology, we intend to fill current evidence gaps, establish standardized evaluation parameters, and offer practical insights for future clinical practice.

## Methods

The methodology for this systematic review and meta-analysis was predefined and registered in PROSPERO (ID: CRD 42024616130). This study complied with the guidelines outlined by the PRISMA (16) (Preferred Reporting Items for Systematic Reviews and Meta-Analyses) and AMSTAR (Assessing the methodological quality of systematic reviews)(**Supplementary Table 1**) (17).

### Search strategy

A thorough and independent literature search was carried out across several primary public databases, including PubMed, Embase, and Web of Science. This search encompassed studies published between January 1996 and November 2024, concentrating on original research concerning lymphedema, lipectomy, and liposuction. The keywords used in the search were “lymphedema”, “lymphoedema”, “lipectomy”, and “liposuction”. Detailed search methodologies, including keywords and Boolean logic combinations, can be found in (**Supplementary Table 2**). Furthermore, beyond the database searches, manual examinations of reference lists and pertinent citations were conducted to uncover potentially eligible studies that may have been missed. During the initial search, no limitations were set aside from the requirement for publications to be in English.

### Study selection

**Figure 1:**
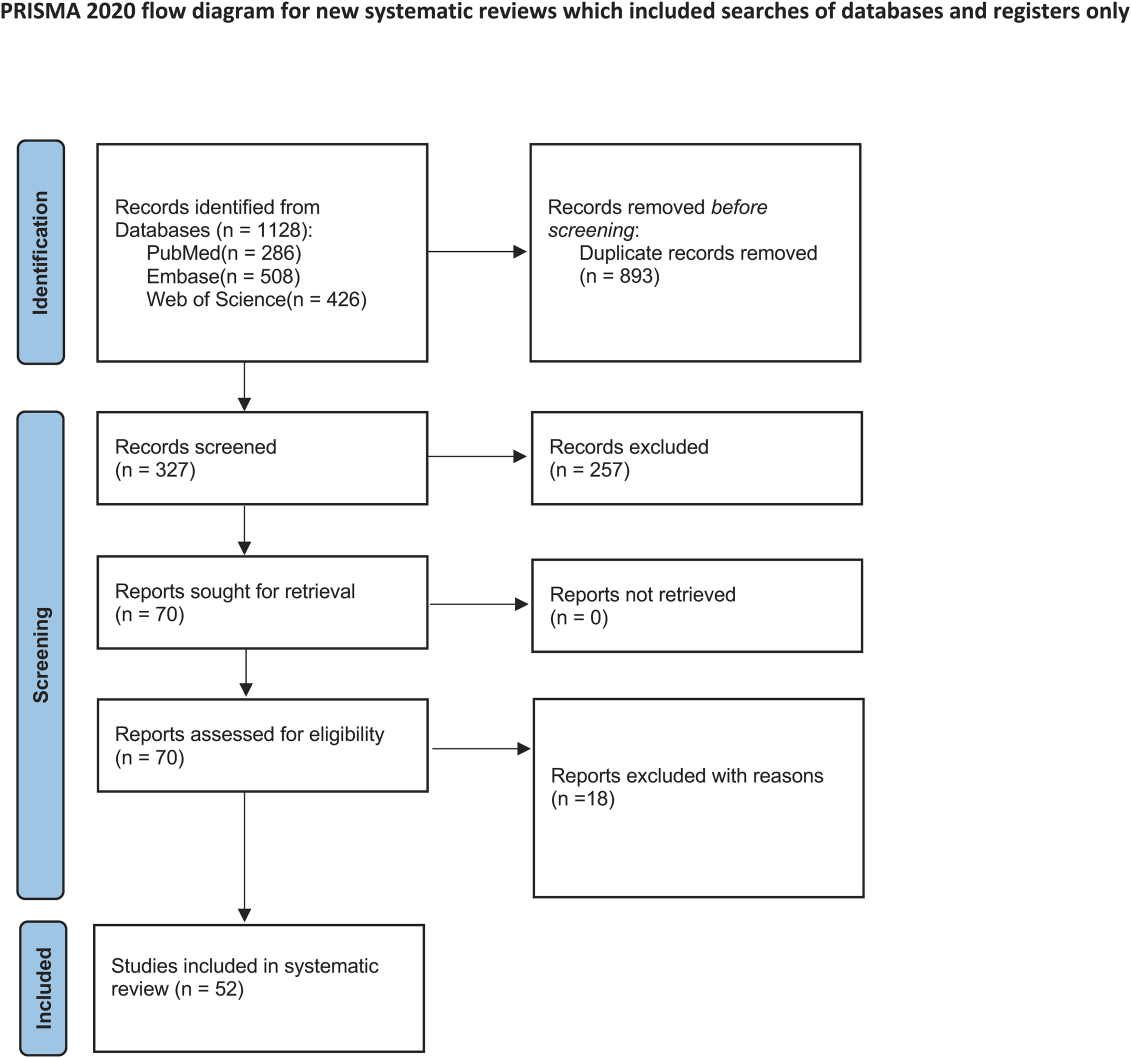
Flow diagram of systematic review.

The study selection process is summarized in **Figure 1**, which follows the PRISMA flow-diagram format. Inclusion Criteria:1. The original studies used randomized controlled trials, prospective cohort studies, retrospective cohort studies, cross-sectional designs, or case series designs. Studies evaluating the outcomes of LS either as a standalone treatment or in combination with physiological procedures such as LVA or VLNT. 3.Studies reporting volume changes and improvement in quality of life as primary outcome measures. 4.Studies with a sample size of at least eight patients. 5.Articles published in English. Exclusion Criteria: 1. Studies focusing solely on surgical treatments other than LS (e.g., VLNT, LVA, RRPP, VLVT, and Charles procedure) without incorporating LS. 2.Studies that did not report relevant outcomes such as volume changes or quality-of-life measures. 3.Review articles, editorials, letters, abstracts, non-original research, and animal studies.

### Data extraction and risk of bias assessment

Data were collected based on the inclusion and exclusion criteria. Two reviewers independently examined all identified articles to ensure a thorough evaluation. In instances where discrepancies arose, the reviewers engaged in discussions to reach agreement. If disagreements continued, a third experienced reviewer was brought in to facilitate mediation and finalize the consensus. Essential information, such as publication year, study design, first author, and surgical techniques, was systematically recorded for each included study. The risk of bias in case series and retrospective studies was assessed using the JBI quality assessment tool for non-controlled trials. Additionally, the Newcastle-Ottawa Scale (NOS) (18) was used to evaluate the quality of the non-controlled trials.

### Statistical analysis

Publication bias was evaluated using Begg’s funnel plot and Egger’s test, with significance set at P < 0.05. To assess the robustness of the pooled results, sensitivity analysis was conducted by iteratively excluding individual studies and observing their impact on the primary outcomes. All statistical analyses were performed using Stata MP version 18.0 (StataCorp). To address multi-arm study designs (e.g., comparisons of upper versus lower extremities or LS combined with technique A versus technique B), data were processed following the Cochrane Handbook for Systematic Reviews of Interventions recommendations. This ensured the avoidance of double counting and the correction for correlated effect sizes. For studies reporting medians with interquartile ranges (IQRs), the means and standard deviations (SDs) were estimated using the method described by Hozo et al. (19). For studies providing only medians and ranges, the method proposed by Wan et al. (20) was used to calculate the equivalent means and SDs. Heterogeneity among studies was quantified using Higgin’s I² statistic and Cochran’s test, which quantifies the proportion of total variation in study estimates owing to heterogeneity, with values ranging from 0% to 100%. Significant heterogeneity was defined as an I² value greater than 50% or a Q-test P-value less than 0.10. In cases of significant heterogeneity, a random effects model was applied to account for between-study variability. When heterogeneity was negligible, a fixed-effects model was used for analysis. Publication bias was evaluated using Begg’s funnel plot and Egger’s test, with statistical significance set at P < 0.05. To assess the robustness of the pooled results, sensitivity analysis was conducted by iteratively excluding individual studies and observing their impact on the primary outcomes. This process tested the stability of the findings and identified potential outliers that could have disproportionately influenced the results.

## Result

### Study selection

The process for selecting studies is depicted in the PRISMA flow diagram (**Figure 1**). An initial search of the databases yielded 1220 entries from PubMed (286), Embase (508), and Web of Science (426), after which 893 duplicates were eliminated. After reviewing the titles and abstracts of the remaining entries, 257 studies were discarded because of their lack of relevance. A full-text eligibility evaluation of 70 studies resulted in the removal of 18 studies for reasons such as insufficient outcome data, absence of pertinent interventions, or flawed study design. Ultimately, 52 studies were incorporated into the systematic review (7, 12, 21–70), and 21 studies involving 929 participants were used in the meta-analysis (**Supplementary Table 3**).

### Quality assessment

The Newcastle-Ottawa Scale (NOS) was used to evaluate the quality of the studies included in the non-randomized studies, while the JBI Critical Appraisal Checklist was utilized for the case series. Details regarding the quality assessment are shown in **Supplementary Table 4**.

### Details of the included studies

A total of 2,334 patients were included in the 52 studies reviewed, with the publication period ranging from 1989 to 2024. The number of patients in each study varied from 10 to 158, resulting in an average of 45.3 patients per study. Sex data were available for 49 of the studies (representing 2,299 patients), revealing that 7.8% were male (179 individuals) and 92.2% were female (2,120 individuals). Anatomical review indicated that 59.3% of the cases involved the upper extremities, 40.3% involved the lower extremities, and 0.4% involved neck lymphedema. The average follow-up was found to be 26.6 months. The study design comprised six prospective cohorts, 14 retrospective cohorts, six prospective case series, and 26 retrospective case series. Regarding surgical techniques, the studies analyzed included 33 that investigated LS as a standalone treatment, 11 examining LS in combination with LVA, 6 studying LS together with VLNT, 4 examining LS along with either VLNT or LVA, 1 that included LS, LVA, and VLNT, and 1 that combined LS with VLVT. The detailed characteristics of all included studies are presented in **Tables 1**, **2**.

**Table 1:** Treatment effect and compression condition of liposuction treatment.

Author	Treatment effect	Postoperative compression	Reference
Lo et al.	30.0 ± 12.3% reduction in affected limb volume.	Post-op compression: 40–50 mmHg for 1 month, then 30–40 mmHg garments.	(21)
Tobias Karlsson et al.	101% average reduction in excess volume at 1 year; 115% at 5 years.	Compression garments worn 24 hours; adjusted based on limb volume changes.	(22)
Manuel E. Cornely et al.	Postoperative limb volume reduction	58.42% patients discontinued CDT after 6 months; 33.82% patients reduced CDT intensity	(23)
W.F. Chen et al.	Average affected limb volume reduction: 32.2%	Short bandaging in the first month, transition to 30–40 mmHg for 1–2 months.	(24)
Tobias Karlsson et al.	Postoperative excess volume: Hand: Reduced by 95% after 1 year, 98% after 5 years. Leg: Reduced by 90% after 1 year, 72% after 5 years	Immediate postoperative compression, long-term compression	(69)
Shuhei Yoshida et al.	Upper limb: 59.3 ± 49.4% reduction; Lower limb: 100.1 ± 37.3% reduction at final follow-up.	Long-term compression: ≥40 mmHg (lower leg), ≥20 mmHg (upper leg/forearm), reduced after 6 months.	(25)
Tobias Karlsson et al.	Median excess volume reduction: 100% (1 year).	Immediate compression post-op; long-term compression required.	(26)
J.M. Lasso et al.	46.2% reduction in limb volume. Satisfaction improved. Improved lymphatic drainage in 17 cases (via SPECT-CT).	Immediate compression; hand: pressure garments after 2 months. Legs: based on tolerance, worn 14 hrs/day. Fifteen patients used elastic garments after 1 year during warm months (once a week); 2 patients did not use garments after this period, while 3 patients needed elastic garments all year, once a week.	(27)
Jianfeng Xin et al.	Pre-op, post-op, and 3-month follow-up excess volumes: 43.2 ± 23.7%, 5.5 ± 12.2%, and 11.6 ± 18.4%, respectively.	Low-tension stockings reduced volume after 6.1 months and stabilized.	(28)
Tobias Karlsson et al.	Mean reduction in excess volume(PG): 106% at 1 year.	Immediate compression; long-term continuous compression garments required.	(29)
C. Chollet et al.	85.4% reduction in excess volume at 10 months. Improved quality of life.	Long-term wear: 25–30 mmHg garments; shorter duration for bandages; garments worn during the day only.	(30)
Melisa D. Granoff et al.	1-year reductions: upper limb: 116%; lower limb: 115%. QoL improved by 33%; cellulitis frequency reduced.	Daytime compression: II–III garments; nighttime III only. 24-hour compression sleeves/gloves.	(31)
Wei F. Chen et al.	Reduced complications (e.g., seroma, hematoma, contour irregularities); improved satisfaction.	Immediate compression; after 5 weeks, 30–40 mmHg compression garments worn lifelong.	(32)
Stewart CJ et al.	Excess volume reductions: 3 months (85%), 1 year (88%), 2 years (94%), 5 years (90%).	Immediate compression post-op, lifelong compression garments, adjusted as needed.	(33)
Hoffner M et al.	Mean excess volume reduction: 117% ± 26% at 5 years.	Immediate compression post-op, 32–40 mmHg compression, garments replaced 6–8 times/year in the first year.	(34)
McGee P et al.	Mean excess volume reductions: 92.6% (6 months), 88.9% (1 year), 113.6% (5 years, 6 patients).	Immediate compression post-op, lifelong compression garments, adjusted as needed.	(35)
Hoffner M et al.	Full reduction achieved at 3 months; sustained during follow-up. Improved QoL scores.	Immediate compression post-op, lifelong 32–40 mmHg compression garments.	(36)
Lamprou DA et al.	Primary lymphedema: 79% excess volume reduction at 2 years. Secondary lymphedema: 101% reduction.	Immediate compression post-op, 24/7 compression garments required long-term.	(37)
Lee D et al.	Cellulitis incidence reduced by 87% (from 0.47/year to 0.06/year). Mean excess volume reduction: 109%.	Immediate compression post-op, lifelong compression garments required.	(38)
Arin K. Greene et al.	Mean excess volume reduction: 73%. Improved QoL, reduced cellulitis episodes.	Immediate compression post-op, new garments fitted after 6 weeks; long-term use required.	(39)
Boyages J et al.	Mean excess volume reduction: 89.6% (arm: 90.2%; leg: 88.2%) at 6 months.	Immediate compression post-op, lifelong compression garments required.	(40)
Jay W. Granzow et al.	Mean excess volume reduction: SAPL:arms:111%, legs:86%;LVA/VLNT: 35% reduction. Cellulitis incidence decreased.	VLNT/LVA: Reduced compression garment use. SAPL: Long-term compression required.	(41)
Mark V. Schaverien et al.	1-year mean excess volume reduction: 101%; maintained at 89% for 5 years.	Lifelong continuous compression garment required.	(42)
S. Mark Taylor et al.	Patients reported improved outcomes in surgical areas.	Elastic bandages post-op; after 1 week, used only at night for 1 month.	(43)
dR J Damstra et al.	118% reduction in excess volume at 12 months.	Immediate compression post-op; customized, tight-fitting compression garments required long-term.	(44)
Brorson H et al.	Patients achieved 109% reduction in excess volume.	Immediate compression post-op; lifelong compression garments required.	(45)
Brorson H et al.	LS+CCT: Mean excess volume reduction: 101%. CCT: Mean reduction: 55%. Improved joint activity and QoL.	Immediate compression post-op; lifelong compression garments required.	(46)
SHIRIN BAGHERI et al.	Excess volume reduction: 76% (2 weeks), 87% (4 weeks), 91% (3 months), 102% (6 months), 109% (1 year).	Immediate compression post-op; lifelong compression garments required.	(47)
Hakan Brorson et al.	LS+CCT: Median excess volume reduction: 115%; CCT: 54%. Lymphatic vessels remained intact post-LS.	LS+CCT: Lifelong compression garments required. CCT: Attempted garment removal led to volume rebound.	(70)
Hakan Brorson et al.	LS+CCT: Mean excess volume reduction: 104%; CCT: 47%.	Lifelong compression garments required; volume rebound noted after garment removal.	(48)
Hakan Brorson et al.	Reduction in excess volume: 87%–97%. Increased skin blood flow. Reduced cellulitis incidence.	Immediate compression post-op; lifelong compression garments required.	(49)
Hakan Brorson et al.	24 patients achieved mean excess volume reduction of 106%.	Immediate compression post-op (32–40 mmHg). Regular replacement and lifelong use required.	(50)
B. McC. O’BRIEN et al.	Mean excess volume reduction: 23% in 10 patients.	Compression garments worn continuously for 6 months, then daytime use only.	(7)
Guido Gabriele et al.	37.9% volume reduction	Long-term pressure garments (grade II-III), 3 sessions of MLD/week for 1 year, tapering later.	(51)
Miaomiao Wei et al.	Median volume reduction: SLNF II: 60.8%, III: 59.8%; SLNF+P II: 56.4%, III: 54.0%; DLNF II: 50.5%, III: 54.4%.	Elastic bandages recommended for at least 6 months.	(52)
Yujin Myung et al.	Volume ratio (affected/healthy): SAL+LVA: 1.06 to 1.35; MSTRAM+VLNT: 1.19→1.26; VLNT: 1.20 to 1.31. LYMPH-Q scores improved.	–	(53)
Xuchuan Zhou et al.	Limb volume reduced; Lymph-ICF-LL scores improved after 12 months of regular CDT.	Compression garments: 30–60 mmHg (day), 20–40 mmHg (night); long-term compression required.	(54)
Kun Chang et al.	Median excess volume reduced from 50.7% to 0.6%.	Immediate compression post-op; garments replaced after 1 month. Majority transitioned to daytime wear only. Most patients (144, 91.1%) only wore compression garments during daytime periodically. Only four (2.5%) needed compression all day long.	(55)
Pedro Ciudad et al.	CRR higher with combined physiological + SAL surgery (85 ± 10.5%). Reduced cellulitis, lower skin tension.	CDT for 5–14 days post-op. Garments reduced at 3 months; 38 patients discontinued garments by 9 months.	(56)
Alina A. Ghazaleh et al.	No significant differences in post-op outcomes, complications, or satisfaction between groups after 24 months.	Not explicitly mentioned in the article.	(57)
Deptula P et al.	Excess volume reduced by 95%; edema reduced by 103% after BB placement.	LS: grade III garments; physiological surgery: limb elevation, then class II compression.	(12)
Alberto Bolletta et al.	Limb circumference reductions: Upper: 80.7 ± 53.7%, Lower: 60.4 ± 32.7%. Cellulitis episodes decreased significantly.	Immediate compression post-op.	(58)
Shuhei Yoshida et al.	Volume reduced; no significant differences between compression types at 6 months post-liposuction.	Bandages + compression stockings more effective within 6 months; pressure: 40 mmHg (calf), 20 mmHg (thigh).	(59)
Brazio, Philip S et al.	All groups achieved an average excess volume reduction of 82% to 106%, maintained for up to 2.4 years. Cellulitis frequency decreased.	Long-term compression; garment levels remained unchanged, but daily compression duration reduced from 12.5 h/d to 7.5 h/d.	(60)
Giuseppe Di Taranto et al.	Mean circumference reduction: Above knee: 52.6 ± 18.9%; Below knee: 42.9 ± 25%; Above ankle: 19.2 ± 34.4%; Foot: 36.2 ± 37%. Reduced limb tension and cellulitis frequency.	Immediate post-op compression garments.	(61)
Pedro Ciudad et al.	Mean circumference reductions: Upper limb: 90%, Lower limb: 85%. Infection rates dropped to zero.	Complex decongestive therapy (CDT) resumed one week post-op, continued for at least 6 months.	(62)
R.G.H. Baumeister et al.	Excess volume reduced from 3417 ± 171 cm³ to 3020 ± 125 cm³ after primary surgery, and to 2516 ± 104 cm³ after secondary surgery.	Post-liposuction: 6 months continuous compression + MLD; 18 patients required no further support treatment thereafter.	(63)
Ida-Maria Leppäpuska et al.	Combined surgery group: 87.7% excess volume reduction; 17 patients reduced garment use; 12 showed improved lymphatic drainage.	Daytime class III garments, nighttime class II for 6 months; reduced compression after 6 months if no swelling.	(64)
Mouchammed Agko et al.	Circumference reduced by 37.9% after VLNT, and by 96.4% after SAL. Infection frequency reduced to zero post-SAL.	Transition to daytime compression after 6.9 months post-SAL; compression discontinued after 10.8 months.	(65)
Corrado Cesare Campisi et al.	Excess volume reduced: Upper: 20.19% to 2.68%; Lower: 21.24% to 2.64%. No infections; lymph flow unaffected.	16 cases (11%) discontinued garments by 12 months; others reduced compression levels.	(66)
Fabio Nicoli et al.	Significant limb volume reduction; skin tension improved by 202%; lymph scintigraphy showed reduced stasis.	Compression garments worn for 4 weeks, then transitioned to nighttime use.	(67)
Fazhi Qi et al.	Circumference reduction in all cases and improved skin softness.	Long-term, regular compression recommended; no specific frequency/load reductions noted.	(68)

**Table 2:** Infection and Qol result of liposuction treatment.

Author	Infection incidence	QoL	Reference
Manuel E. Cornely et al.	16.92% reported up to 12 recurrent erysipelas per year before surgery. After surgery, no patient had recurrent erysipelas during the observation period.	92.3% LYMQOL improvement.	(23)
W.F. Chen et al.	–	LYMQOL scores showed statistically significant improvements in appearance (P = 0.019), function (P = 0.046), and symptoms (P = 0.014). Emotional improvement was observed but did not reach statistical significance (P = 0.052). Overall quality of life improved significantly (P = 0.033).	(24)
Tobias Karlsson et al.	Pre-op: 0.20/person/year (52% prevalence); Post-op: 0.07/person/year (23% prevalence). Reduced by 65%.	-	(26)
J.M. Lasso et al.	5 patients reported three per year or more cellulitis episodes/year pre-op; no post-op cellulitis reported.	improvement in quality of life after the procedure with an increase in overall satisfaction of five mean points in a 20point survey (range 2–10 points of increase). The improvements in “satisfaction with limb appearance” were remarkable, with a 2.1 mean increase.	(27)
Jianfeng Xin et al.	20 cases of post-op cellulitis; 42 cases reported no recurrence.	The feeling of heaviness and fatigue in the operated limb was alleviated by the 3-month follow-up compared with that preoperatively, whereas feelings of stiffness, tenderness, and tightness worsened. There were no significant differences in pain, numbness, or weakness between preoperative and 3-month follow-ups	(28)
C. Chollet et al.	–	QoL improved in the physical, psychological and social health domains QoL was evaluated with the EQ-5D scale and the Upper Limb Lymphedema	(30)
Melisa D. Granoff et al.	Ninety-two episodes of cellulitis were reported in our patient cohort prior to debulking over a total of 348.5 disease years (0.26 episodes/year). In comparison, 2 episodes of cellulitis were reported after debulking over the course of 27.4 post-operative years (0.07 episodes/year).	LYMQOL sub-scores improved on all metrics for patients with upper extremity LE, with the largest improvement in the Appearance sub-score (44%)LYMQOL sub-scores improved on all metrics for patients with lower extremity LE, with the largest improvement in the Appearance sub-score (37%)	(31)
Stewart CJ et al.	Pre-op: 21 cellulitis cases; Post-op: 3 recurrences.	–	(33)
McGee P et al.	-	Patient reported quality of life outcomes improved in the ten patients who completed LyQLi questionnaires (**Figure 2**). Pre-operatively the mean LyQLi score was 75.9 (range 29–111) which improved to a mean of 26.9 at 12 month follow-up	(35)
Hoffner M et al.	–	SF-36 QOL: reduced pain/emotional scores, improved general health, mental health, and social function. Compared with SF-36 norm data for the Swedish population, only physical functioning showed lower values than the norm at baseline. After liposuction, general health, bodily pain, vitality, mental health, and social functioning showed higher values at various time points.	(36)
Lamprou DA et al.	Before surgery patients with primary lymphoedema had a mean of 8 attacks of cellulitis of the leg each year. This decreased to 0·2 attacks per year after the procedure(P < 0·001). Respective numbers in patients with secondary lymphoedema were 6 attacks per year before CSAL which reduced to 0·3 attacks per year (P < 0·001).	-	(37)
Lee D et al.	Cellulitis frequency: 0.47/year pre-op to 0.06/year post-op (87% reduction).Erysipelas incidence dropped significantly (p<0.001) from 0.47 attacks/year (range 0-5.0, SD 0.8 attacks/year) to 0.06 attacks/year (range 0-3.0, SD 0.3 attacks/year) after liposuction, a reduction of 87%.	–	(38)
Arin K. Greene et al.	75% reduction in cellulitis risk.	-	(39)
Boyages J et al.	–	Improvements in pain, heaviness, self-consciousness, anxiety, swelling, and emotional impact. Functionally, all patients reported improvements on the PSFS index of personally important activities by 6 months post-surgery (p \ 0.01). Improvements were also evident in the standardized domains of pain, heaviness, self-consciousness, levels of anxiety, perceived degree of swelling, and emotional impact; such improvements were statistically significant, with the exception of pain in the lower limb and anxiety about the upper limb.	(40)
Jay W. Granzow et al.	Pre-op cellulitis incidence: 70%, post-op: 10%.	-	(41)
Mark V. Schaverien et al.	–	Anxiety: 9.09 to 4.6; Depression: 5.73 to 1.70; VAS: 64.6 to 81.2,anxiety and depression scores improved.	(42)
Brorson H et al.	-	Visual scoring.	(46)
Hakan Brorson et al.	Cellulitis frequency: 0.4/person/year pre-op to 0.1/person/year post-op.	-	(48)
B. McC. O’BRIEN et al.	Pre-op: cellulitis in 7 patients; Post-op: 3 recurrences.	–	(7)
Guido Gabriele et al.	Lymphangitis reduced from 4.6/year/person pre-op to 0.95/year/person post-op.	LLIS improved from 68.7 pre-op to 16 post-op.	(51)
Miaomiao Wei et al.	Higher cellulitis rate observed in Stage III patients, except for DLNF (Stage II).	The median postoperative overall QOL score for stage II patients were observed to be 8 for the SLNFþP group, 8 for the SLNF group, and 7 for the DLNF group. In contrast, the preoperative median scores were 6, 6, and 6, respectively. Postoperative assessments revealed that, in stage III patients, the median QOL scores were 7 in the SLNFþP group,7 in the SLNF group, and 8 in the DLNF group. These scores showed an improvement from the preoperative median scores of 5.5, 6, and 6, respectively	(52)
Yujin Myung et al.	Cellulitis cases reduced from 5 pre-op to 2 post-op.	LYMQOL improved from 67 to 43 post-op. From the LYMPH-Q questionnaire scores, we observed in all three groups that the discomfort felt by the patients due to lymphedema postoperatively was significantly reduced compared with that preoperatively. The MSTRAM + VLNT group showed the greatest degree of improvement; their preoperative LYMPH-Q scores decreased from an average of 68 points to 22 points at 12 months post-surgery (P < 0.01). In the other two groups, the LYMPH-Q score significantly decreased (67–43 and 70–50, respectively).	(53)
Xuchuan Zhou et al.	–	Lymph-ICF-LL scores improved significantly. In the CDT-compliant group (Group B), scores decreased from 51.21 preoperatively to 36.99 at 12 months postoperatively. In the non-compliant group (Group A), scores decreased from 55.08 preoperatively to 49.53 at 12 months postoperatively	(54)
Kun Chang et al.	84.8% of patients had no cellulitis; others had rare recurrences (0.6% had up to 6 episodes/year).	-	(55)
Pedro Ciudad et al.	Stage II: 3→0.5/year; Stage III: 4→0.8/year (p <.01).	–	(56)
Alina A. Ghazaleh et al.	-	On a numeric rating scale from zero to 10 with zero representing the highest and 10 the lowest level of satisfaction, no significant difference could be found for the level of patient satisfaction following VLNT or VLNT + WAL: the mean patient satisfaction in the VLNT group was 1.80 (SD = 0.80) versus 1.40 (SD = 0.70) in the VLNT + WAL group (p = 0.323).	(57)
Alberto Bolletta et al.	Cellulitis: 1.4 ± 1.9 to 0.1 ± 0.4 episodes/year.	–	(58)
Brazio, Philip S et al.	Of 11 patients with a cellulitis history, 10 experienced no further recurrences.	-	(60)
Giuseppe Di Taranto et al.	Cellulitis frequency reduced to 0.1 ± 0.3 episodes.	–	(61)
Pedro Ciudad et al.	Number of episodes of infection Upper limb lymphedema 0.8 to 0, lower 1.4 to 0	-	(62)
Ida-Maria Leppäpuska et al.	47.6% had pre-op cellulitis; reduced to 14.3% post-op.	–	(64)
Mouchammed Agko et al.	Pre-op: 1.8 infections/year; post-VLNT: 1; post-SAL: 0.	-	(65)
Corrado Cesare Campisi et al.	No infections reported during 6-month follow-up.	–	(66)
Fazhi Qi et al.	The onsets of erysipelas were average 6.45 times per year in the patients before surgical treatment. In 10 patients, erysipelas did not recur within 1 year after surgery. Only one patient had erysipelas recurrence at 10 months after operation. Four patients had one erysipelas recurrence at the 2nd year after surgery. Two patients had twice, and three patients had one erysipelas recurrence at the 3rd year after surgery.	-	(68)

### Outcome analysis

#### Change in excess volume

**Figure 2:**
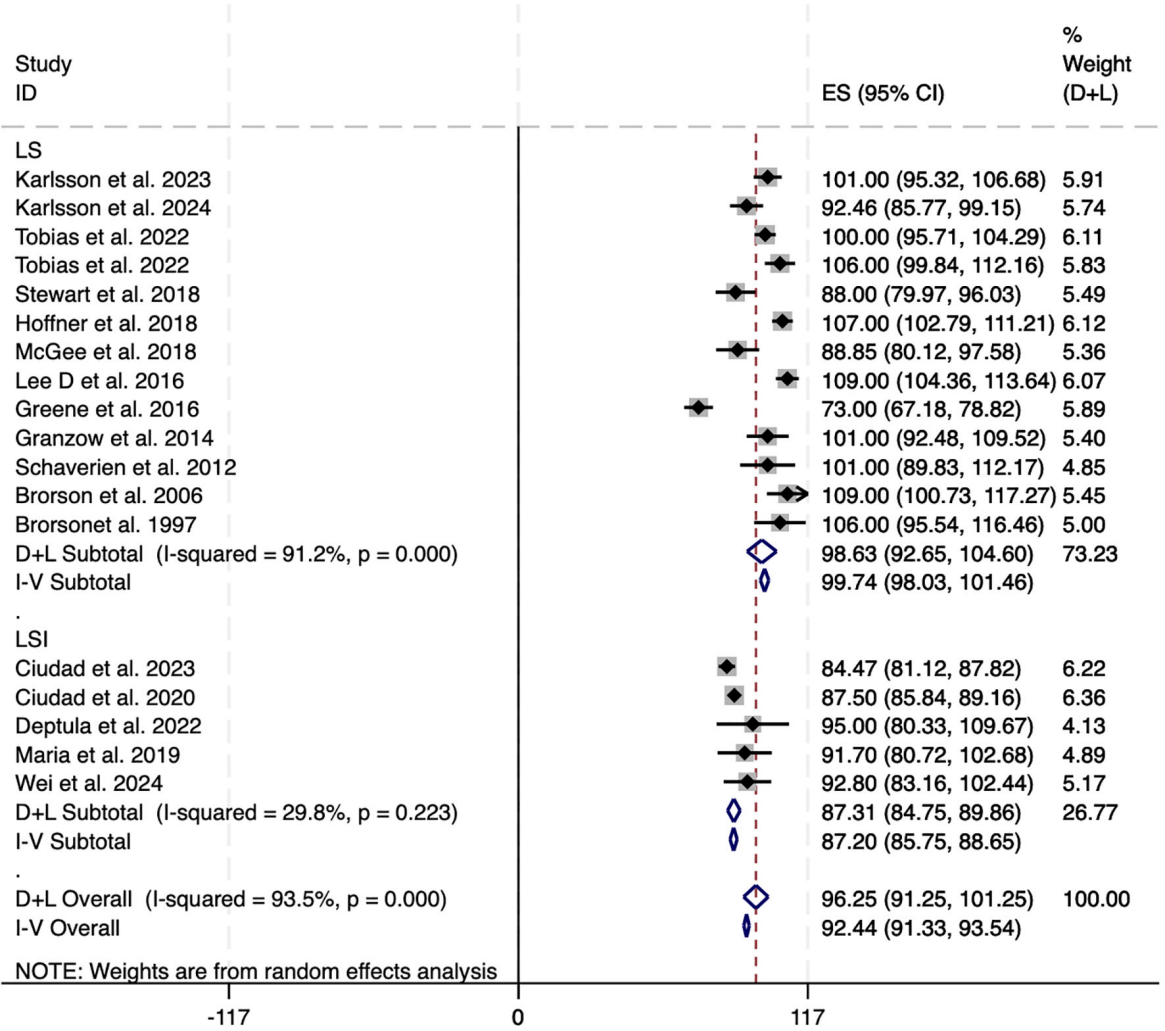
Forest plots of excess circumference reduction in LS and LSI. CI, confidence interval; LS, Liposuction; LSI, Liposuction integrated surgery.

Among the 25 studies that documented alterations in excess volume(affected limb volume − healthy limb volume)/healthy limb volume × 100%), 17 (encompassing 798 patients) provided adequate data for inclusion in the meta-analysis. Of these 17 investigations, two examined LS in conjunction with LVA or VLNT, one explored LS combined with LVA, and two focused on LS paired with VLNT. Studies were excluded if they did not provide sufficient statistical details or reported only medians without ranges or interquartile ranges (IQRs). A random-effects model was employed because of the significant heterogeneity (I² = 93.2%). The overall reduction in excess circumference across the 17 studies was 92.44% [95% CI: 91.33 to 93.5] (**Figure 2**). In the subgroup analysis, LS alone resulted in nearly complete reduction in excess volume (99.74%, 95% CI: 98.03 to 101.46; I² = 91.2%). Conversely, LS combined with physiological interventions yielded slightly lower pooled reductions (87.31%, 95% CI: 84.75 to 89.86; I² = 29.8%), but showed decreased heterogeneity (**Figure 2**). The diminished effect size and reduced heterogeneity observed with LSI therapies might be attributable to variations in the combination of interventions used.

**Figure 3:**
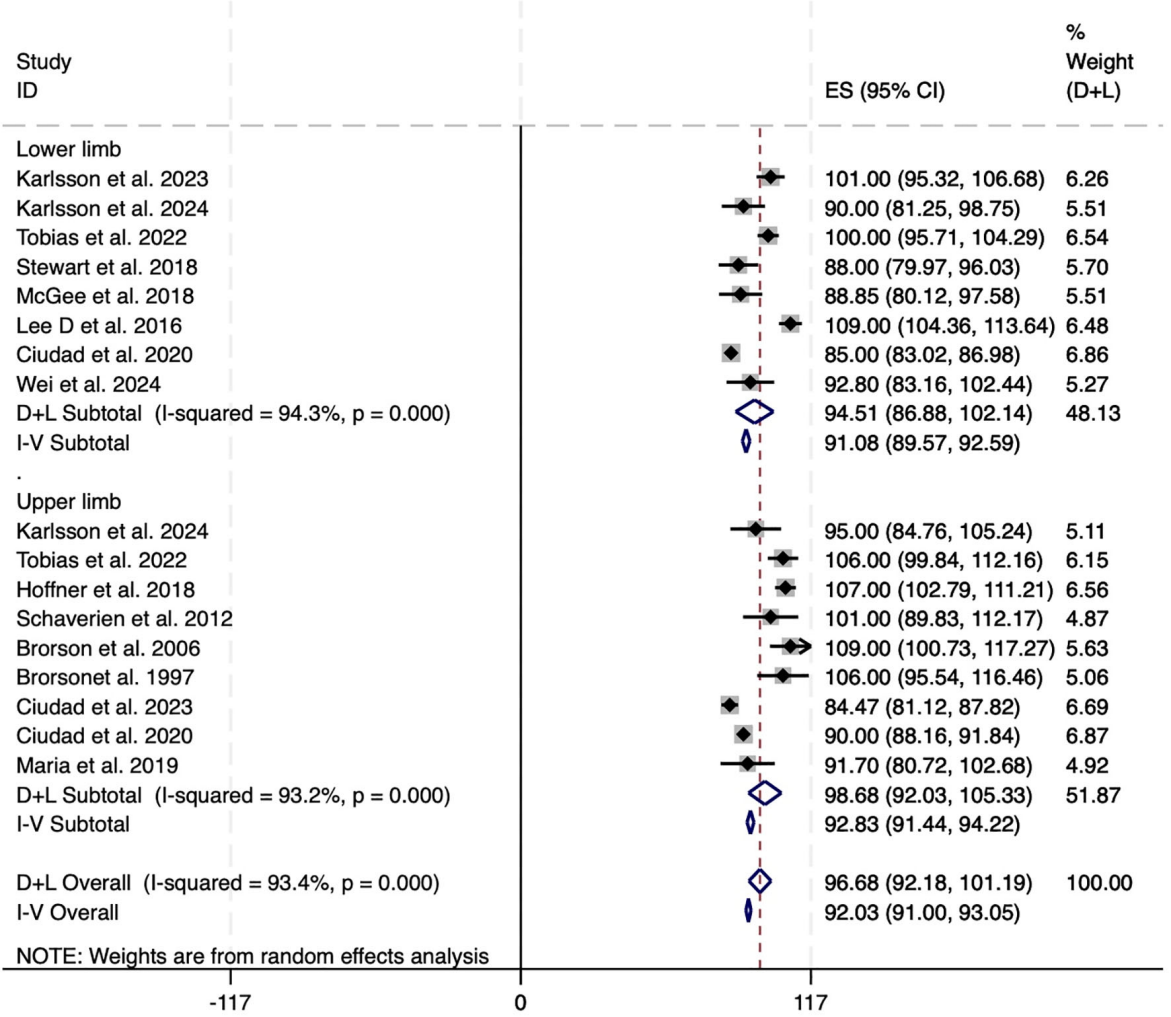
Forest plots of excess circumference reduction in upper and lower limb. CI, confidence interval; LS, Liposuction; LSI, Liposuction integrated surgery.

Additionally, subgroup analysis based on the affected limb location revealed no major disparity in treatment efficacy between upper and lower limb cases. Pooled reduction in excess volume for lower limb lymphedema was 91.08% (95% CI: 89.57 to 92.59; I² = 94.3%), while that for upper limb cases was slightly higher at 92.83% (95% CI: 91.44 to 94.22; I² = 93.2%). These results suggest that LS may be similarly effective in managing both upper and lower limb lymphedema, though substantial heterogeneity remained across studies (**Figure 3**).

### Improvement in infection episodes

**Figure 4:**
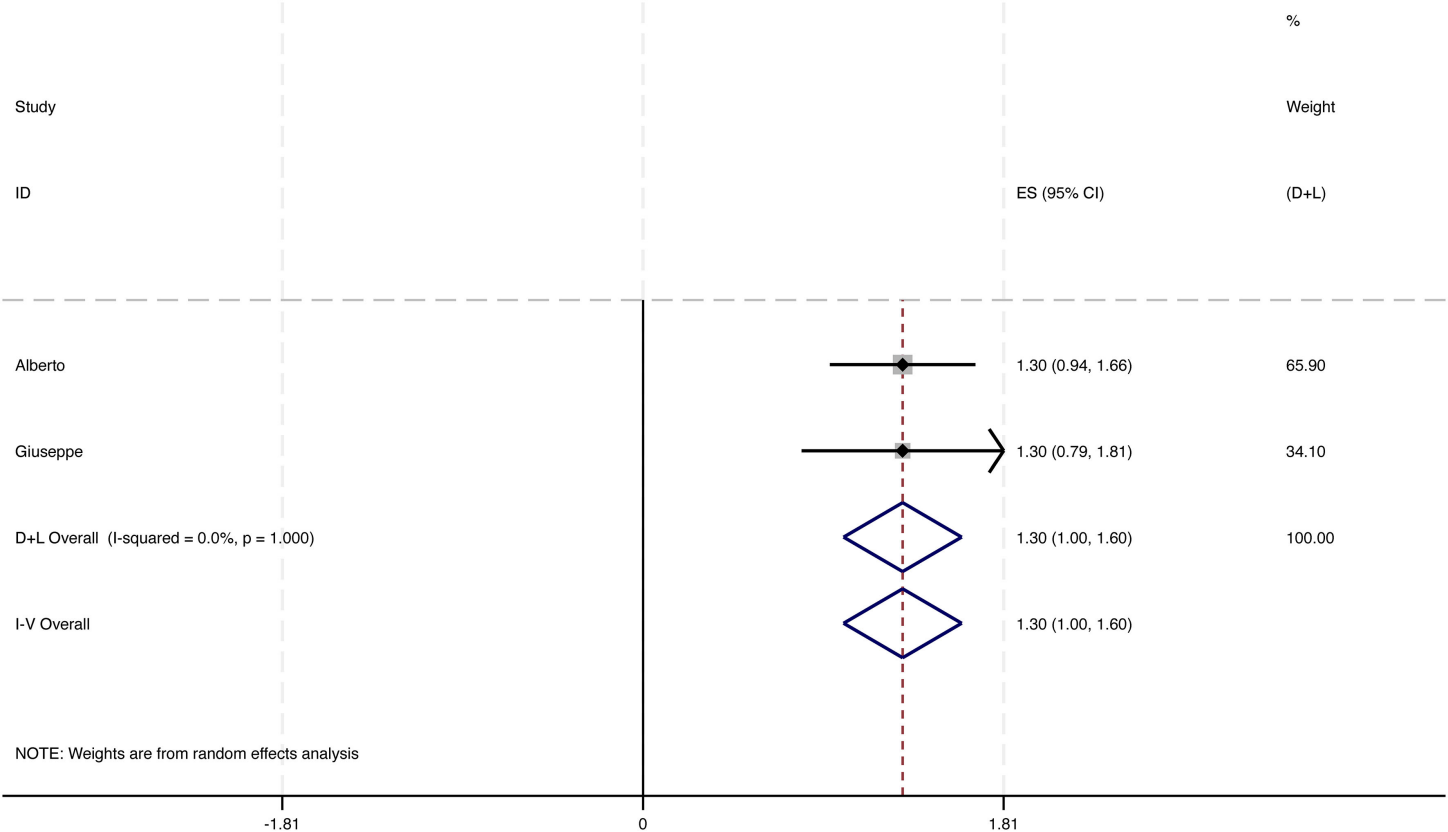
Change in number of infectious episodes per year.

Fewer episodes of skin infections were observed in 24 of the 52 studies, while 20 of these studies supplied adequate data regarding annual changes in infection occurrences. Among these, 4 studies encompassing a total of 339 patients were included in the meta-analysis. The overall decrease in the frequency of infection episodes was found to be 0.95 [95% CI: 0.85 to 1.05], accompanied by notable heterogeneity. Following the removal of two outlier studies, the recalculated pooled reduction among 131 patients was 1.30 [95% CI: 1.00 to 1.60], exhibiting low heterogeneity (I² = 0%) (see **Figure 4**). This underscores the potential of interventions based on LS to significantly decrease infection rates in individuals with lymphedema. These findings should be interpreted cautiously given the limited number of eligible studies and variability in definitions and follow-up.

### Publication bias

The possibility of publication bias was evaluated using Egger’s and Begg’s tests, neither of which indicated significant evidence of bias. Visual inspection of the funnel plots revealed symmetrical distributions, which further validated the strength of the findings.

### Compression dependence

Across the included studies, reliance on compression following liposuction-based interventions was heterogeneously defined and reported. Therefore, we synthesized these outcomes narratively and present study-level details in **Table 1**. In general, LS maintained a requirement for long-term compression to preserve volume control, whereas combined approaches were consistently associated with a reduction in compression burden. Specifically, multiple cohorts reported down-titration of garment class/pressure and fewer daily wearing hours after combined procedures, with a proportion of patients discontinuing compression entirely. These patterns were observed across both upper- and lower-extremity lymphedema, although between-study variability in definitions, follow-up windows, and perioperative protocols precluded a single pooled estimate.

### Quality of life

Quality of life outcomes were assessed using validated instruments but varied across studies (LYMQOL, LLIS, SF-36 and LyQLi), limiting quantitative aggregation. We therefore provide a structured narrative synthesis and detailed tabulation in **Table 2**. Overall, LS-based interventions were associated with clinically meaningful improvements from baseline, most commonly in appearance, symptoms, function and emotional domains. Studies employing combined procedures frequently demonstrated sustained gains at mid- to longer-term follow-up, aligning with reduced compression dependence and improved limb function.

appearance (P = 0.019), function (P = 0.046), and symptoms

(P = 0.014). Emotional improvement was observed but did not

reach statistical significance

## Discussion

This meta-analysis provides comprehensive insights into the effectiveness of treatments based on LS for lymphedema, highlighting significant reductions in limb volume, reliance on compression therapy, and frequency of infection episodes. Standalone LS effectively reduces the volume almost completely by directly removing fibrotic and hypertrophic adipose tissues. When combined with techniques such as VLNT or LVA, LSI enhances lymphatic regeneration and drainage restoration, thereby alleviating both physical and immune-related challenges. However, comparisons indicate that LSI achieves slightly less volume reduction than standalone LS. These results primarily support our initial hypothesis that LSI is less effective than standalone LS in achieving volume reduction within a short time frame. There are two key reasons for this discrepancy. First, LSI techniques require the preservation of additional tissue to perform LVA or VLNT, which is essential for maintaining blood flow and sustaining functional lymphatic structures in the affected area (53–56). Consequently, the total amount of tissue removed was less than that in standalone LS. Second, VLNT results in a gradual decline over several months, dependent upon recovery of the lymphatic drainage system. Therefore, the relatively short follow-up periods in these studies may not adequately capture the long-term outcomes of the volume reduction.

Despite these variations, LSI offers significant advantages by reducing dependence on compression therapy and enhancing long-term QOL. Although earlier research indicated that lymphangiogenesis occurs following LS treatment, the immediate effects of LS remain incompletely understood (71). A primary limitation of LS is its reliance on continuous compression therapy to maintain results. By contrast, as illustrated in **Tables 1**, **2**, the integration of LS with physiological procedures provides distinct benefits by substantially reducing the need for postoperative compression therapy. This improvement is further corroborated by the restoration of lymphatic function, which contributes to enduring outcomes. Analyses of subgroups revealed that a notable proportion of patients undergoing LS in conjunction with LVA or VLNT achieved complete cessation of compression therapy within 3–12 months, with most of approximately 6 months (64–66). Among the remaining patients, the majority experienced significant reductions in both pressure and frequency of compression garment use. Importantly, these combined strategies resulted in marked improvements in the QOL scores, demonstrating enhanced functional outcomes and reduced physical burden.

Recent advances in liposuction techniques reflect both procedural refinements and expanded conceptual roles within combined surgical strategies. Initially limited to ISL stage II due to concerns about its efficacy in fibrotic limbs, liposuction has evolved significantly with the introduction of power-assisted liposuction (PAL) (7, 72). This innovation—using vibrating cannulas—has demonstrated the ability to partially disrupt fibrotic and adipose tissues, thereby enhancing fat removal efficiency and reducing surgical time. As a result, PAL has broadened practical use in fibrotic limbs; however, effectiveness varies and appears to depend on strict compression adherence and careful case selection, with limited high-certainty evidence in advanced fibrosis (51, 73–75). This broader applicability has been supported by clinical evidence, with some studies documenting sustained volume reduction lasting up to 15 years. Moreover, incorporating agents such as hyaluronidase into tumescent solutions has improved tissue compliance and reduced the duration of postoperative compression therapy, further facilitating enhanced recovery and long-term outcomes (23). However, since LS alone does not restore lymphatic drainage, it is increasingly performed in combination with LVA or VLNT to achieve more lasting outcomes. The optimal combination strategy should be tailored to disease characteristics. Given the risk of damaging residual lymphatics, particularly in late-stage disease, selective liposuction techniques have emerged. These involve sparing lymphatic-rich areas identified by preoperative indocyanine green (ICG) mapping (56, 62, 66). LS and physiological reconstructions may be performed in a single session or staged depending on the lymphatic architecture. Some studies suggest that LVA remains effective with deep lymphatic system, when superficial lymphatic structures are not preserved, allowing for more aggressive fat removal where necessary. In patients lacking functional lymphatics, LS combined with VLNT is generally preferred (52, 58, 65, 67, 76, 77). Triple-combination strategies (LS + LVA + VLNT) have shown potential superiority over dual combinations in reducing limb volume, skin tension, and infection recurrence (61). However, robust comparative data remain limited, and high-quality randomized controlled trials are needed to establish clear indications and refine procedural sequencing. The choice between single-stage and staged approaches remains an area of active debate. Staged procedures offer the advantage of targeting dominant disease components—starting with LS for solid-predominant cases or physiological surgery for fluid-predominant ones (78). One-stage surgery may be preferable in mixed presentations or when minimizing hospitalizations is a priority (60). Nonetheless, performing multiple procedures in the same anatomical area poses challenges: LVA requires intact superficial lymphatics, while VLNT depends on preserved vascular supply and minimal postoperative compression. Segmental or compartment-based strategies and standardized surgical protocols are essential to mitigate these risks and improve outcomes.

Importantly, this meta-analysis revealed that LS-based interventions demonstrated similarly high efficacy in both upper and lower limb lymphedema. Despite prior concerns that gravitational effects and tissue characteristics might lead to weaker responses in the lower extremities, subgroup analysis showed nearly equivalent volume reductions—91.08% for lower limb cases and 92.83% for upper limb cases. These findings suggest that LS-based surgical approaches, whether standalone or combined with physiological techniques, can be broadly effective across anatomical locations. This reinforces the clinical versatility of LS and supports its application as a robust intervention for managing lymphedema, regardless of limb involvement.

Infection presents a significant challenge in individuals with lymphedema. Our systematic review demonstrated that LS-based treatment markedly decreased the occurrence of episodes of infection. In the standalone LS group, the removal of inflammatory tissue resulted in a notable reduction in the recurrence of infections compared with the preoperative baseline, which aligns with previous research findings. Patients undergoing LSI also exhibited a considerable decline in infection rates, likely due to enhanced lymphatic drainage and immune system modulation through VLNT and LVA. However, it is essential to recognize that the limited number of studies adhering to the criteria for meta-analysis poses challenges in establishing definitive comparisons between LS and LSI. Among the studies that met the eligibility criteria, only two were suitable for meta-analysis, revealing a small yet clinically significant decrease in approximately one episode of infection following surgery. This highlights the need for future well-designed studies that use standardized reporting and consistent statistical techniques to further substantiate these promising findings.

This research offers several advantages, including a comprehensive examination of 52 studies involving 2,357 patients and a primary focus on volume reduction, compression therapy reliance, and infection rates. The subgroup analyses provided valuable insights into the comparative benefits of standalone LS versus LSI, elucidating their distinct clinical applications (**Tables 1**, **2**). However, this study had several limitations. The notable heterogeneity observed in standalone LS studies (I² = 91.2%) reflects the variability in surgical techniques, patient demographics, and postoperative care protocols. Differences in follow-up duration and adherence to compression therapy likely influenced the results, underscoring the need for standardized methodologies (79, 80). Specifically, future studies should: (1) adopt a unified set of primary outcomes that includes a standardized definition of excess volume reduction; reports cellulitis as events per patient-year; specifies explicit metrics for “compression dependence” (e.g., garment class/pressure in mmHg and hours of wear per day); and uses validated quality-of-life instruments (e.g., LYMQOL, LLIS, or SF-36 at pre-specified timepoints); (2) standardize assessment windows at 3, 6, 12, 24, 36, and 48 months postoperatively; (3) stratify and report outcomes by ISL stage (II vs. III) and by limb (upper vs. lower); and (4) predefine and comprehensively report perioperative compression protocols (bandaging sequence, garment class/pressure, duration) as well as surgical parameters (e.g., PAL vs. SAL, cannula diameter, and tumescent composition including the hyaluronidase dose). Additionally, several of the included studies employed retrospective designs, which may introduce risks associated with selection and reporting biases. Moreover, to harmonize outcomes across studies, we converted medians (with IQRs or ranges) to means and standard deviations using established methods; while widely accepted, these transformations can introduce minor imprecision. The limited sample sizes of LSI therapy studies further constrain the generalizability of the findings. Finally, the long-term outcomes remain inadequately understood, particularly regarding the sustainability of volume reduction and the implications of discontinuing compression therapy, highlighting the necessity for future longitudinal research.

To address these deficiencies, future studies should prioritize standardization of methods that include consistent outcome measures and follow-up protocols. Conducting large-scale randomized controlled trials(RCTs) are essential to validate the effectiveness of lymphatic system interventions and establish clinical guidelines based on robust evidence. The absence of high-quality RCTs in the current literature limits strong causal inferences regarding treatment outcomes, as most studies rely on retrospective case series. Therefore, future research in this field should focus on filling this critical gap. Furthermore, additional research should explore the complex relationship between restoration of lymphatic drainage and volume reduction to enhance patient outcomes. Additionally, investigations should assess the long-term benefits of decreasing compression dependence, particularly in comprehensive procedures, in order to deepen the overall understanding of treatment effectiveness.

## Conclusion

This systematic review and meta-analysis validated the efficacy of liposuction-based therapies in reducing limb volume and may reduce incidence of infection among patients with lymphedema. Independent LS results in a significant immediate decrease in volume, whereas the combination of LS with physiological approaches, such as LVA or VLNT, enhances long-term outcomes by addressing the underlying causes of lymphatic dysfunction. Future research should focus on improving the methodological consistency, exploring the long-term benefits of combined treatments, and establishing standardized clinical protocols to optimize patient outcomes and therapeutic strategies.

## Data Availability

The datasets presented in this study can be found in online repositories. The names of the repository/repositories and accession number(s) can be found in the article/**Supplementary Material**.

## Supplementary material

Supplementary Table 1 AMSTAR 2.

Supplementary Table 2 Search strategy.

Supplementary Table 3 Basic information of the studies.

Supplementary Table 4 Quality information of the studies.
