# Supplementary material for: Outcomes after liposuction-based treatment of lymphedema: a systematic review and meta-analysis
Source: Front Oncol. 2025 Nov 26;15:1651472. doi: 10.3389/fonc.2025.1651472 (PMC12689340; doi:10.3389/fonc.2025.1651472)
Supplement: Supplementary Table 2 — Search strategy. [file Table2.docx]

Pubmed

("Lipectomy"[Mesh] OR "Lipectomies" OR "Lipoplasty" OR "Lipoplasties" OR "Aspiration Lipectomy" OR "Aspiration Lipectomies" OR "Lipectomies, Aspiration" OR "Lipectomy, Aspiration" OR "Lipolysis, Suction" OR "Suction Lipolysis" OR "Liposuction" OR "Liposuctions" OR "Suction Lipectomy" OR "Lipectomies, Suction" OR "Lipectomy, Suction" OR "Suction Lipectomies" OR "Aspiration Lipolysis" OR "Lipolysis, Aspiration") AND ("lymphedema"[Mesh] OR "Lymphedemas" OR "Milroy Disease" OR "Congenital Hereditary Lymphedema" OR "Congenital Hereditary Lymphedemas" OR "Hereditary Lymphedema, Congenital" OR "Hereditary Lymphedemas, Congenital" OR "Lymphedema, Congenital Hereditary" OR "Lymphedemas, Congenital Hereditary" OR "Early Onset Lymphedema" OR "Early Onset Lymphedemas" OR "Lymphedemas, Early Onset" OR "Hereditary Lymphedema" OR "Hereditary Lymphedemas" OR "Lymphedema, Hereditary" OR "Lymphedemas, Hereditary" OR "Lymphedema, Early-Onset" OR "Early-Onset Lymphedema" OR "Early-Onset Lymphedemas" OR "Milroy's Disease" OR "Milroys Disease" OR "Nonne-Milroy Disease" OR "Nonne Milroy Disease" OR "Nonne-Milroy Lymphedema" OR "Lymphedema, Nonne-Milroy" OR "Nonne Milroy Lymphedema" OR "Nonne-Milroy-Meige Disease" OR "Nonne Milroy Meige Disease" OR "Primary Congenital Lymphedema" OR"Congenital Lymphedema, Primary" OR "Congenital Lymphedemas, Primary" OR "Lymphedema, Primary Congenital" OR "Lymphedemas, Primary Congenital" OR "Primary Congenital Lymphedemas" OR "Congenital Familial Lymphedema" OR "Hereditary Lymphedema Type I")

Embase

('lymphedema' OR 'lymphedemas' OR 'milroy disease' OR 'congenital hereditary lymphedema' OR 'hereditary lymphedema' OR 'early onset lymphedema' OR 'nonne disease' OR 'meige disease') AND ('lipectomy' OR 'lipectomy' OR 'lipectomies' OR 'lipoplasty' OR 'lipoplasty' OR 'lipoplasties' OR 'aspiration lipectomy' OR 'liposuction' OR 'suction lipectomy')

WOS

TS=(ipectomy OR lipectomies OR lipoplasty OR lipoplasty OR lipoplasties OR aspiration lipectomy OR liposuction OR suction lipectomy) AND TS=(lymphedema OR lymphedemas OR milroy disease OR congenital hereditary lymphedema OR hereditary lymphedema OR early onset lymphedema OR nonne disease OR meige disease)
