# Supplementary material for: Outcomes after liposuction-based treatment of lymphedema: a systematic review and meta-analysis
Source: Front Oncol. 2025 Nov 26;15:1651472. doi: 10.3389/fonc.2025.1651472 (PMC12689340; doi:10.3389/fonc.2025.1651472)
Supplement: Supplementary Table 3 — Basic information of the studies. [file Table3.docx]

| Author | Year | Study Type | Country | NOS | JBI |
| --- | --- | --- | --- | --- | --- |
| Lo et al. | 2024 | RCS | USA |  | 1/1/1/1/1/1/1/0/1/1=9/10 |
| Tobias Karlsson et al. | 2023 | PC | Sweden | 1/0/1/1/1/1/1/1=7 |  |
| Manuel E. Cornely et al. | 2023 | RCS | Germany |  | 1/1/1/1/1/1/1/0/0/1=8/10 |
| W.F. Chen et al. | 2023 | RC | China |  | 1/1/1/1/1/1/1/0/1/1=9/10 |
| Tobias Karlsson et al. | 2023 | RC | Australia | 1/0/1/1/1/0/1/1=6 |  |
| Shuhei Yoshida et al. | 2023 | RC | Japan | 1/0/1/1/1/1/1/1=7 |  |
| Tobias Karlsson et al. | 2022 | RCS | Sweden |  | 1/1/1/1/1/1/1/0/0/1=8/10 |
| J.M. Lasso et al. | 2022 | RCS | Spain |  | 1/1/1/1/1/1/1/0/1/1=9/10 |
| Jianfeng Xin et al. | 2022 | RCS | China |  | 1/1/1/1/1/0/1/0/0/1=7/10 |
| Tobias Karlsson et al. | 2022 | RCS | Sweden |  | 1/1/1/1/1/1/1/0/1/1=9/10 |
| C. Chollet et al. | 2021 | PCS | France |  | 1/1/1/1/1/1/1/0/1/1=9/10 |
| Melisa D. Granoff et al. | 2021 | RCS | USA |  | 1/1/1/1/1/1/1/1/1/1=10/10 |
| Wei F. Chen et al. | 2019 | RC | USA | 1/0/1/1/1/1/1/1=7 |  |
| Stewart CJ et al. | 2018 | RCS | UK |  |  |
| Hoffner M et al. | 2018 | PCS | Sweden |  | 1/1/1/1/1/1/1/0/1/1=9/10 |
| McGee P et al. | 2018 | PCS | UK |  | 1/1/1/1/1/1/1/0/1/1=9/10 |
| Hoffner M et al. | 2017 | RC | Sweden | 1/0/1/1/0/1/1/1=6 |  |
| Lamprou DA et al. | 2016 | RCS | Netherlands |  |  |
| Lee D et al. | 2016 | PC | Sweden | 1/0/1/1/0/1/1/1=6 |  |
| Arin K. Greene et al. | 2016 | RCS | USA |  | 1/1/1/1/1/1/1/0/1/1=9/10 |
| Boyages J et al. | 2015 | PC | Australia |  |  |
| Jay W. Granzow et al. | 2014 | RC | USA | 1/0/1/1/0/1/1/1=6 |  |
| Mark V. Schaverien et al. | 2012 | PCS | UK |  | 1/1/1/1/1/1/1/0/1/1=9/10 |
| S. Mark Taylor et al. | 2009 | RCS | Canada |  | 1/1/1/0/1/1/1/0/0/1=7/10 |
| dR J Damstra et al. | 2009 | PCS | Netherlands |  | 1/1/1/1/1/1/1/0/1/1=9/10 |
| Brorson H et al. | 2006 | RCS | Sweden |  | 1/1/1/1/1/1/1/0/1/1=9/10 |
| Brorson H et al. | 2006 | RCS | Sweden |  | 1/1/1/1/1/1/1/0/1/1=9/10 |
| SHIRIN BAGHERI et al. | 2005 | RCS | Sweden |  | 1/1/1/1/1/1/1/0/1/1=9/10 |
| Hakan Brorson et al. | 1998 | PCS | Sweden |  | 1/1/1/1/1/1/1/0/1/1=9/10 |
| Hakan Brorson et al. | 1998 | RCS | Sweden | 1/0/1/1/0/1/1/1=6 |  |
| Hakan Brorson et al. | 1997 | RCS | Sweden |  | 1/1/1/1/1/1/1/0/1/1=9/10 |
| Hakan Brorson et al. | 1997 | RCS | Sweden |  | 1/1/1/1/1/1/1/0/1/1=9/10 |
| B. McC. O’BRIEN et al. | 1989 | RCS | Australia |  | 1/1/1/1/0/1/1/0/1/1=8/10 |
| Guido Gabriele et al. | 2024 | RCS | Italy |  | 1/1/1/1/1/1/1/0/1/1=9/10 |
| Miaomiao Wei et al. | 2023 | RC | China | 1/0/1/1/0/1/1/1=6 |  |
| Yujin Myung et al. | 2023 | RC | South Korea | 1/0/1/1/0/1/1/1=6 |  |
| Xuchuan Zhou et al. | 2023 | RC | China | 1/0/1/1/0/1/1/1=6 |  |
| Kun Chang et al. | 2023 | RCS | China |  | 1/1/1/1/1/1/1/0/1/1=9/10 |
| Pedro Ciudad et al. | 2023 | RCS | Italy |  | 1/1/1/1/1/1/1/0/1/1=9/10 |
| Alina A. Ghazaleh et al. | 2022 | PC | Switzerland | 1/0/1/1/0/1/1/1=6 |  |
| Deptula P et al. | 2022 | RC | Switzerland | 1/0/1/1/0/1/1/1=6 |  |
| Alberto Bolletta et al. | 2022 | RC | China | 1/0/1/1/0/0/1/1=5 |  |
| Shuhei Yoshida et al. | 2021 | RC | Japan | 1/0/1/1/0/1/1/1=6 |  |
| Brazio, Philip S et al. | 2022 | RC | USA | 1/0/1/1/0/1/1/1=6 |  |
| Giuseppe Di Taranto et al. | 2021 | PC | China | 1/0/1/1/0/1/1/1=6 |  |
| Pedro Ciudad et al. | 2020 | RC | Peru | 1/0/1/1/0/1/1/1=6 |  |
| R.G.H. Baumeister et al. | 2020 | RCS | Germany | 1/1/1/1/1/1/1/0/1/1=9/10 |  |
| Ida-Maria Leppäpuska et al. | 2019 | RCS | Finland |  | 1/1/1/1/1/1/1/0/1/1=9/10 |
| Mouchammed Agko et al. | 2017 | PC | China | 1/0/1/1/0/1/1/1=6 |  |
| Corrado Cesare Campisi et al. | 2017 | RCS | Italy |  | 1/1/1/1/1/1/1/0/1/1=9/10 |
| Fabio Nicoli et al. | 2015 | RCS | China |  | 1/1/1/1/1/1/1/0/1/1=9/10 |
| Fazhi Qi et al. | 2009 | RCS | China |  | 1/1/1/1/1/1/1/0/1/1=9/10 |

PC:prospective cohort; RC;retrospective cohort; PCS; prospective case series; RCS retrospective case series
