# Supplementary material for: Outcomes after liposuction-based treatment of lymphedema: a systematic review and meta-analysis
Source: Front Oncol. 2025 Nov 26;15:1651472. doi: 10.3389/fonc.2025.1651472 (PMC12689340; doi:10.3389/fonc.2025.1651472)
Supplement: Supplementary Table 4 — Quality information of the studies. [file Table4.docx]

| Author | Sex | Age  (year) | Classification/ Limb | Duration of disease  (month) | | Stage | Method | Follow-up  (month) |
| --- | --- | --- | --- | --- | --- | --- | --- | --- |
| Lo et al. | Female:33 male: 3 | 57.82 | P:19, S:17 | - | - | | LS + CDT | 12 |
| Tobias Karlsson et al. | Female:59 male: 8 | 50 | PL:36, SL:31 | 144 | | ISL Stage II-III | LS + CCT | 60 |
| Manuel E. Cornely et al. | Female: 65 | - | S:65 | 48 | | ISL Stage II–III | PAL + TLA + H + CDT | 180 |
| W.F. Chen et al. | Female:53 male: 2 | 55.95 | U:23, L:34 | 159.6 | | ISL Stage I–III | PAL | 10.7 |
| Tobias Karlsson et al. | Female:54 male: 5 | 57 | SU:29, PL:12, SL:18 | U:84, L: 168 | | ISL Stage II–III | LS + CCT | 60 |
| Shuhei Yoshida et al. | Female:25 male: 3 | 61.5 | U:10, L:18 | - | | - | LS + CDT | 17.4 |
| Tobias Karlsson et al. | Female:110 male: 14 | 49 | PL:63, SL:61 | 132 | | - | LS + CCT | 60 |
| J.M. Lasso et al. | Female:18 male: 2 | 52.4 | SU:12, SL:5, PU:1, PL:2 | - | | ISL Stage II | ICG-guided LS | 12 |
| Jianfeng Xin et al. | Female:46 male: 16 | 55.6 | SL:62 | 55.8 | | ISL Stage II–III | LS + CCT | 3 |
| Tobias Karlsson et al. | Female: 18 | 61 | SU:18 | 108 | | ISL Stage II–III | LS + CCT | 12 |
| C. Chollet et al. | Female: 14 | 56.5 | SU:14 | - | | ISL Stage II | LaB + CDT | 20.5 |
| Melisa D. Granoff et al. | Female:34 male: 7 | 58.1 | U:23, L:18 | 106.8 | | - | PAL + CDT | 12 |
| Wei F. Chen et al. | Female:36 Male:5 | 56.4 | mLIPO: U:12, L:3, sLIPO: U:18, L:8 | - | | ISL Stage II–III | mLIPO:26, sLIPO:15 | 20 |
| Stewart CJ et al. | Female:65 male: 7 | 45.6 | PL:42, SL:30 | 228 | | ISL Stage II–III | LS + CCT | 108 |
| Hoffner M et al. | Female: 105 | 64 | SU:105 | 120 | | ISL Stage II | LS + CCT | 60 |
| McGee P et al. | Female: 21 | 52 | SL:21 | 182.4 | | ISL Stage II–III | LS + CCT | 96 |
| Hoffner M et al. | Female: 60 | 64 | SU:60 | 120 | | ISL Stage II | LS + CCT | 12 |
| Lamprou DA et al. | Female:67 male: 21 | 47.5 | PL:47, SL:41 | PL: 240 , SL: 144 | | ISL Stage III | CASL + CCT | 24 |
| Lee D et al. | Female: 130 | 63 | SU:130 | 105.6 | | - | LS + CCT | 6 |
| Arin K. Greene et al. | Female:12 male: 3 | 45 | SU:6, SL:9 | - | | - | LS + CCT | 37.2 |
| Boyages J et al. | Female: 21 | 55.7 | SU:15, SL:6 | SU: 109.2, SL: 186 | | ISL Stage II–III | LS + CCT | 12 |
| Jay W. Granzow et al. | Female: 26 | 57 | SU:26, LVA:8, VLNT:8, SAPL:10 | LVA: 99.6, VLNT: 45.6, SAPL: 154.8 | | ISL Stage I–III | LVA; VLNT; SAPL | LVA: 27 VLNT: 32, SAPL: 12 |
| Mark V. Schaverien et al. | Female: 12 | 48 | SU:12 | 84 | | - | LS + CCT | 60 |
| S. Mark Taylor et al. | - | - | SHN:10 | - | | - | LS + CCT | 6 |
| dR J Damstra et al. | Female: 37 | 48 | SU:37 | 98.4 | | ISL Stage II | CASL + CCT | 12 |
| Brorson H et al. | Female: 11 | 49 | SU:11 | 108 | | ISL Stage II–III | LS + CCT | 6 |
| Brorson H et al. | Female: 49 | 48 | SU:49 (LS+CCT:35, CCT:14) | LS+CCT: 88.8, CCT: 94.8 | | ISL Stage II | LS + CCT, CCT | 12 |
| SHIRIN BAGHERI et al. | Female: 20 | 64 | SU:20 | 132 | | - | LS + CCT | 12 |
| Hakan Brorson et al. | Female: 20 | - | SU:20 (LS+CCT:11, CCT:9) | LS+CCT: 90 , CCT: 85.2 | | ISL Stage II | LS + CCT, CCT | 12 |
| Hakan Brorson et al. | Female: 28 | - | SU:28 (LS+CCT:14, CCT:14) | LS+CCT: 93.6, CCT: 94.8 | | ISL Stage II | LS + CCT, CCT | 12 |
| Hakan Brorson et al. | Female: 12 | 62 | SU:12 | 84 | | - | LS + CCT | 12 |
| Hakan Brorson et al. | Female: 28 | 63 | SU:28 | 84 | | - | LS + CCT | 12 |
| B. McC. O’BRIEN et al. | Female:16 male: 3 | 50 | 19 (P:5, S:14; U:10, L:9, D:2) | 132 | | - | LS + CCT | 9.5 |
| Guido Gabriele et al. | Female:22 Male:2 | 58 | U:20, L:4 | 67.92 | | Campisi Stage: II: 1, III: 18, IV: 5 | Concurrent sLVA + LS | 12 |
| Miaomiao Wei et al. | Female:35 | 55 | SL:35 | 15 | | ISL Stage II–III | SLNF+P+LS, SLNF+LS, DLNF+LS | 15 |
| Yujin Myung et al. | Female:87 | 52.3 | SU:87 | 97.2 | | ISL Stage IIb–III | SAL + LVA, MSTRAM + VLNT + LVA, Gastroepiploic VLNT + LVA | 12 |
| Xuchuan Zhou et al. | Female:55 | 54.33 | L:55 | 40 | | ISL Stage I–III | LVA + LS + CDT | 12 |
| Kun Chang et al. | Female:158 | 57 | SU:158 | 12 | | ISL Stage II–III | LS + LVA | 12 |
| Pedro Ciudad et al. | Female:78 | 49.4 | 78 | 39.2 | | ISL Stage II–III | LVA, Gastroepiploic VLNT, Gastroepiploic VLNT + DIEP, SAL + LVA | 26.4 |
| Alina A. Ghazaleh et al. | Female:97 | - | SU:97 | - | | ISL Stage II:88, III:5, 2 cases unclassified | Groin VLNT + WAL | 24 |
| Deptula P et al. | - | 62 | 14 | - | | ISL Stage II–III | LS + LVA/VLNT + BB; Concurrent LS + physiological surgery; LS + LVA/VLNT + BB | - |
| Alberto Bolletta et al. | Female:71 Male:23 | 50 | U:83, L:11 (P:10, S:84) | - | | ISL Stage IIb:74, III:20 | Gastroepiploic VLNT + SAL; SAL performed 12.0 ± 2.4 days after VLNT | 36 |
| Shuhei Yoshida et al. | Female:18 Male:1 | 59.6 | SL:13, P:6 | - | | ISL Stage II–III | Primary: LVA or Groin VLNT; Secondary: LS + CCT | 15.3 |
| Brazio, Philip S et al. | Female:16 Male:5 | 56.3 | P:2, S:19 | - | | ISL Stage I–III | LS + Physiological Surgery: Simultaneous Surgery; LS first, then Physiological Surgery; Physiological Surgery first, then LS; Physiological Surgery only | 28.8 |
| Giuseppe Di Taranto et al. | Female:29 Male:8 | 51.7 | SL:37 | - | | ISL Stage II–III | Gastroepiploic VLNT + LS; Gastroepiploic VLNT + LVA + LS(Liposuction performed 2 weeks after primary VLNT or LVA) | 24 |
| Pedro Ciudad et al. | Female:22 Male:2 | 54.5 | UL:12, LL:12 | 28 | | ISL Stage IIb–III | VAL + LVA | 12 |
| R.G.H. Baumeister et al. | Female:28 | 56.3 | SU:28 | - | | ISL Stage III | Lymph Vessel Transplantation + Secondary Liposuction | 37 |
| Ida-Maria Leppäpuska et al. | Female:48 | 56.7 | SU:48 | 52 | | ISL Stage II–III | Groin LN + LS | 27–71 |
| Mouchammed Agko et al. | Female:12 | 52 | SU:6, SL:6 | 32 | | ISL Stage II | Dual Gastroepiploic VLNT + SAL | 23.5 |
| Corrado Cesare Campisi et al. | Female:107 Male:39 | - | 146 (PU: 20, SU: 43, PL: 49, SL: 34) | U: 19.3, L: 18.9 | | Campisi Stage: IIB–IIIB | FLLA-LVSP + CCT | 12 |
| Fabio Nicoli et al. | Female:10 | 54.6 | SU:10 | - | | ISL Stage II | Supraclavicular OR Groin Free LN Flap, followed by laser-assisted liposuction | 6 |
| Fazhi Qi et al. | - | - | SU:11 | 96 | | - | LS + Latissimus Dorsi Flap + Fascia Transplant | 12–36 |

MLD:manual lymphatic drainage; LVA:lymphatico-venous anastomosis; VLNT:vascularized lymph node transfer; SLVA: Supremicrosurgical Lymphatico-Venular Anastomosis; SCIP: superficial circumflex iliac perforator; FLLA-LVSP: fibro-lipo-lypmh-aspiration with a lymph vessel sparing procedure; LN: lymph node; VAL: VASER-assisted MSTRAM: autologous breast reconstruction with muscle-sparing transverse rectus abdominis muscle flap; SC-VLNT:lipectomy supraclavicular vascularized lymph node transfer; SLNF+P:single lymph nodes flap with a skin paddle; DLNF:dual lymph nodes flap without a skin paddle; BB:BioBridge; Primary Lymphedema：P，Secondary Lymphedema：S，upper limb：U，lower limb：L，Head and Neck：HN，Dual：D，LS : liposuction； TLA+H: The adapted tumescent solution +hyaluronan; LELI: SAL :Suction-assisted lipectomy ; SAPL: Suction assisted protein lipectomy ; CSAL: Circumferential suction-assisted lipectomy; VAL : Vaser-assisted liposuction; LaB :Liposuction-assisted brachioplasty; PAL: Power-Assisted Liposuction; CDT : complex (or complete) decongestive therapy; CCT: controlled compression therapy; mLIPO : Modified liposuction with skin excision ; sLIPO: standard liposuction without skin excision
